# Translation and validation of the Portuguese version of the Keratoconus Outcomes Research Questionnaire (KORQ)

**DOI:** 10.5935/0004-2749.20210067

**Published:** 2021

**Authors:** Roberto Damian Pacheco Pinto, Flávia Cid Gomes, Carlos Eduardo Leite Arieta, Monica Alves

**Affiliations:** 1 Discipline of Ophthalmology and Otorhinolaryngology, Faculdade de Ciências Médicas, Universidade Estadual de Campinas, Campinas, SP, Brazil

**Keywords:** Keratoconus, Cornea, Quality of life, Questionnaire, Corneal ectasia, Ceratocone, Cornea, Qualidade de vida, Questionário, Ectasia corneana

## Abstract

**Purpose:**

The purpose of this study is to translate and validate a Portuguese version of the Keratoconus Outcomes Research Questionnaire. The Keratoconus Outcomes Research Questionnaire is a psychometrically robust and valid instrument used to assess the impact of keratoconus on activity limitations and symptoms.

**Methods:**

We performed a translation, cross-cultural adaptation, and validation of the Portuguese version of the Keratoconus Outcomes Research Questionnaire. The initial translation of the English version to the Portuguese language was performed by two independent native speaker translators, followed by an interdisciplinary panel evaluation of the translated version. The Portuguese version was then back-translated into English by two independent native speakers, followed by evaluation and comparison with the original English version by the same interdisciplinary panel. For subsequent validation, the translated questionnaire was administered at two different times to a population of 30 subjects, and the results were compared in a concordance analysis.

**Results:**

The translation into Portuguese and back-translation were determined to be correct. Thirty participants were enrolled in the study (mean age, 29.23 ± 7.56 years). Nine questions (31%) had almost perfect agreement (questions 3, 4, 5, 8, 18, 22, 27, 28, and 29), 15 questions (51.7%) had substantial agreement (questions 1, 2, 6, 7, 9, 12, 14, 15, 16, 17, 20, 21, 23, 25, and 26), 4 questions (13.8%) had moderate agreement (questions 10, 11, 19, and 24) and 1 question (3.5%) had reasonable agreement (question 13). High-correlation coefficients were obtained when comparing results of the initial application and second application of this questionnaire to a sample of 30 individuals, which indicated excellent concordance with regard to results, repeatability, and reliability.

**Conclusions:**

This translated and validated questionnaire can be applied to a larger population with the intent to assess quality of life in keratoconus patients in the overall Brazilian population as well as in distinct regions of the country.

## Abstract (pt)

**Objetivo:**

Desenvolver a versão em Português do Keratoconus Outcomes Research Questionnaire (KORQ). O Keratoconus Outcomes Research Questionnaire é um instrumento psicometricamente válido e robusto para avaliação do impacto do ceratocone na limitação de atividades e sintomas.

**Métodos:**

Foi realizado no estudo a tradução, adaptação transcultural e validação em Português do Keratoconus Outcomes Research Questionnaire. A tradução inicial da versão em inglês para o idioma português foi realizada por dois tradutores de língua nativa inglesa independentes, seguida de uma avaliação interdisciplinar da versão traduzida. Após isso, a versão em Português foi traduzida novamente para o inglês por dois tradutores nativos de língua portuguesa independentes, seguida de avaliação e comparação com a versão original em inglês pelo mesmo painel interdisciplinar. Para a subsequente validação, o questionário traduzido foi aplicado em dois tempos diferentes em uma população de 30 indivíduos, e os resultados foram comparados em uma análise de concordância.

**Resultados:**

O processo de tradução para a língua portuguesa e tradução reversa do questionario Keratoconus Outcomes Research Questionnaire foi conduzido de maneira satisfatória. Trinta participantes foram incluídos no estudo (média idade, 29.23 ± 7.56 anos). Nove questões (31%) com concordância quase perfeita (questões 3, 4, 5, 8, 18, 22, 27, 28 e 29), cinco questões (51.7%) com concordância substancial (questões 1, 2, 6, 7, 9, 12, 14, 15, 16, 17, 20, 21, 23, 25 e 26), quatro questões (13.8%) com concordância moderada (questões 10, 11, 19 e 24) e uma questão (3.5%) com concordância razoável (questão 13). Os altos coeficientes de correlação obtidos ao comparar os resultados da aplicação inicial com a re-aplicação do questionário em uma amostra de 30 indivíduos indicam a excelente concordância em relação aos resultados, repetibilidade e confiabilidade.

**Conclusão:**

Esse questionário traduzido e validado pode ser aplicado em populações maiores com o objetivo de avaliar a qualidade de vida em pacientes com ceratocone na população brasileira em geral, assim como em regiões distintas do país.

## INTRODUCTION

Keratoconus is a progressive corneal disease with an onset that typically occurs in adolescence or early adulthood^(1)^. It is a degenerative disorder of the eye in which structural changes in the cornea cause thinning, development of a conical shape, and, in most advanced cases, corneal scarring^(2)^. Optical aberrations and degradation of visual performance can profoundly impair visual function, severely affecting an individual’s day-to-day activities and overall quality of life (QoL)^(3)^. Treatment of keratoconus consists of spectacles, contact lenses, implantation of intrastromal corneal ring segments, and corneal collagen crosslinking^(4)^; when these treatments are no longer effective, lamellar or penetrating keratoplasty can be considered^(5)^. However, surgical intervention may lead to significant QoL implications, such as slow and lengthy postsurgical recovery, extended time off work, high risk of postsurgical complications, secondary complications related to long-term steroid use (e.g., cataract, glaucoma), recurrence of keratoconus, residual refractive errors, and so forth^(6)^. Even nonsurgical therapeutic options such as spectacles or contact lenses can affect QoL because of their negative effects on cosmesis or handling difficulties^(7-9)^.

Symptom questionnaires have been increasingly implemented to assess the QoL related to a specific disease, quantify symptoms, evaluate natural disease courses, and determine the impact of treatment strategies. Generic or ophthalmic patient-reported outcome (PRO) measures, not specific to keratoconus, may not in clude essential items to capture unique, keratoconus-specific QoL issues^(10,11)^. Nevertheless, because there is no keratoconus-specific PRO instrument, some studies have investigated the impact of keratoconus on patient QoL using PRO instruments developed for other conditions (e.g., cataract or refractive error)^(12)^. However, a psychometrically robust and valid instrument has recently been developed to assess the impact of keratoconus on activity limitations and symptoms, called the Keratoconus Outcomes Re search Questionnaire (KORQ)^(13)^. Using standard protocols for instrument development, the KORQ was developed in the early 2000s, and it was first published in 2007 and redeveloped in 2016. The KORQ is currently the only validated keratoconus-spe cific PRO measure^(13)^. A previous study evaluated the psychometric properties of the KORQ using both classical test theory and Rasch analysis and concluded that the KORQ is a psychometrically robust PRO measure for the evaluation of QoL parameters in individuals with keratoconus and is appropriate for use in both clinical and research settings^(14)^.

The widespread use of questionnaires in distinct populations warrants translated and language-adapted versions. To expedite this goal, many translation guidelines have been published that have attempted to ensure the equivalence between the original and translated versions^(15,16)^.

This study aimed to validate the Portuguese-language version of the KORQ, with the goal of creating a relevant tool that will facilitate a better understanding of the impact of keratoconus on QoL in the Brazilian population.

## METHODS

The KORQ comprises two scales: the Activity Limitation scale with 18 items and the Symptoms scale with 11 items. Each item is rated on a four-point scale with an additional “not applicable” option. The patient’s score is obtained through ready-to-use Microsoft Excel scoring (available at http://links.lww.com/OPX/A287 and http://links.lww.com/OPX/A288) spreadsheets for the two scales of the KORQ. When the study sample is similar to the original study (i.e., the same inclusion and exclusion criteria we used in this study, detailed below), these spreadsheets can be used to convert respondents’ raw scores into person measures in logits without having to perform Rasch analysis. Each spreadsheet consists of three sheets labeled as ‘‘rawdata,’’ ‘‘raschscore,’’ and ‘‘raw to Rasch conversion.’’ Users are required to register respondents’ responses to items using a numerical label (i.e., 1 to 4) in the ‘‘rawdata’’ sheet, and the corresponding Rasch scores automatically appear in the ‘‘raw to rasch conversion’’^(13)^. The inclusion criteria included a diagnosis of keratoconus or history of penetrating keratoplasty for keratoconus and age >18 years. Patients who had a significant level of other comorbid ocular conditions (severe glaucoma, uveitis history, retinal diseases with visual impairment, cataract), who had undergone ocular surgery other than for keratoconus, who had any significant systemic disease, or who were unable to read Portuguese and understand the KORQ were excluded.

The present study was conducted in the Department of Ophthalmology and Otorhinolaryngology at the University of Campinas and had a transversal, observational, and noninterventional design. It was performed after approval was obtained from the local research ethics committee (CAAE: 86636418.4.0000.5404) and was conducted in accordance with the tenets of the Declaration of Helsinki and current legislation on clinical research. Written informed consent was obtained from all subjects after they were provided with an explanation of the procedures and study requirements. We followed a three-phase process initially proposed by Beaton and Gjersing to obtain a scientifically accurate translation and transcultural validation of the original English version of the KORQ into the target Portuguese-language version^(15,16)^. First, the initial translation of the English version into the Portuguese language was performed by two independent native speaker translators, followed by an interdisciplinary panel evaluation of the translated version. Second, two independent native speakers back-translated the Portuguese version into English, followed by evaluation and comparison with the original English version by the same interdisciplinary panel. Third, the final version of the KORQ was applied to a selected population to evaluate observer concordance. Our validation process, as listed below, was based on this guideline. The following tasks were completed:

1. Two native Portuguese speakers translated the original English language of the KORQ into the Portuguese language.
2. An interdisciplinary committee, composed of three ophthalmologists (two general ophthalmologists and one corneal specialist) and two residents, evaluated both the Englishand Portuguese-language versions to ensure an adequate translation and transcultural adaptation without altering the applicability of the questionnaire.
3. Two native English speakers back-translated the final Portuguese version questionnaire receiving after committee approval.
4. The interdisciplinary committee reevaluated the back-translated file in comparison with the original.
5. The Portuguese-language questionnaire was applied to a sample of 30 individuals at distinct time points with an interval of two days.

**Figure 1:**
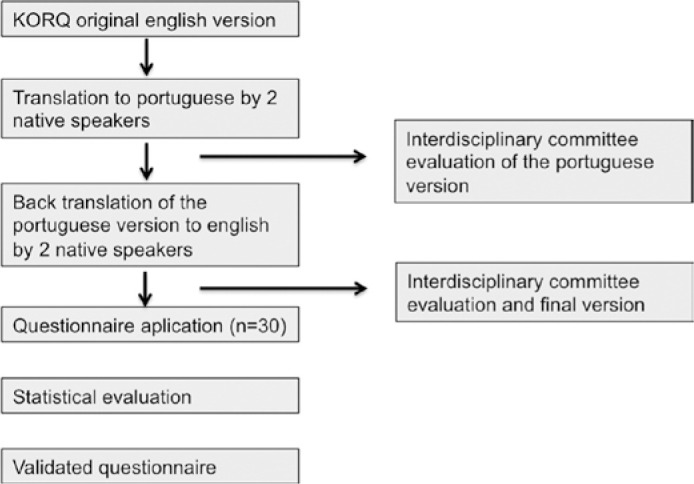
Translation and validation of the Keratoconus Outcomes Research Questionnaire (KORQ) into Portuguese: study design.

Data were analyzed using the STATA 14.0 program (StataCorp LP, College Station, TX, USA). Frequency tables were used for descriptive analysis. The Kappa agreement method was applied to all questions for the interpretation of the coefficients considered: reasonable agreement for values between 0.20-0.39, moderate agreement for values between 0.40-0.59, agreement between 0.60-0.79, and almost perfect agreement between 0.80-1.00. We used the Z test to assess the significance of the Kappa coefficient. For all tests, a p value <0.05 was considered as significant. Figure 1 summarizes the study design.

## RESULTS

The KORQ Portuguese version was applied twice to a population comprising 30 patients aged 19 to 45 years (mean age, 29.23 ± 7.56 years). Because the questionnaire comprised simple and direct queries, no difficulties emerged during the translation or adaptation steps. Similarly, the remaining steps of the process did not raise any controversies. The translated and validated questionnaire is shown in table 1 (functional) and table 2 (symptoms).

**Table 1:** KORQ questionnaire in Portuguese: Part I-Activity Limitation

1. Quanto sua visão interfere com o uso de uma tela de computador?
2. Quanto sua visão interfere para dirigir durante o dia?
3. Quanto sua visão interfere para dirigir durante a noite?
4. Quanto sua visão interfere com a leitura de sinais de trânsito?
5. Quanto sua visão interfere em assistir TV?
6. Quanto sua visão interfere em subir ou descer degraus?
7. Quanto sua visão interfere em evitar objetos no seu caminho?
8. Quanto sua visão interfere na sua capacidade de fazer seu trabalho?
9. Quanto sua visão interfere em enxergar a distância?
10. Quanto que luzes em sua direção interferem com sua habilidade de enxergar para realizar suas tarefas?
11. Quanto sua visão interfere em fazer tarefas para perto?
12. Quanto sua visão interfere em fazer o seu hobby (passatempo)?
13. Quanto sua visão interfere em reconhecer rostos faces?
14. Quanto sua visão interfere em enxergar com pouca luz?
15. Quanto sua visão interfere em fazer tarefas domésticas? (ex. Limpar, passar roupa, lavar)
16. Quanto sua visão interfere para identificar profundidade das coisas?
17. Quanto sua visão interfere para enxergar objetos pequenos a longas distâncias? (por exemplo: pipas, aviões no céu)
18. Quanto sua visão interfere em atividades de observação? (ex. Câmera, microscópio, binóculos, etc)

KORQ= Keratoconus Outcomes Research Questionnaire.

**Table 2:** KORQ questionnaire in Portuguese: Part II-Symptoms

1. Quanto você se sente incomodado com visão distorcida?
2. Quanto você se sente incomodado por ofuscamento e necessidade de usar óculos escuros o tempo todo?
3. Quanto que um dia ensolarado interfere na sua capacidade de enxergar, de fazer suas tarefas?
4. Quanto você se sente incomodado com o uso de lentes de contato rígidas?
5. Quanto você se sente incomodado com dores de cabeça decorrentes do uso de óculos ou lentes de contato?
6. Quanto você se sente incomodado por sintomas de olho seco?
7. Quanto você se sente incomodado em dias com muito vento?
8. Quanto você se sente incomodado quando está cansado?
9. Quanto você se sente incomodado em dias com ar seco?
10. Quanto você se sente incomodado em dias com poeira?
11. Quanto você se sente incomodado em locais com poluição no ar?
7. Quanto você se sente incomodado em dias com muito vento?
8. Quanto você se sente incomodado quando está cansado?
9. Quanto você se sente incomodado em dias com ar seco?
10. Quanto você se sente incomodado em dias com poeira?
11. Quanto você se sente incomodado em locais com poluição no ar?

KORQ= Keratoconus Outcomes Research Questionnaire.

Table 3 shows the Kappa values and respective confidence intervals and p values for each questionnaire item. There was significant interobserver agreement between the two measures for all questions (p<0.05). With regard to the classification of agreement, 9 questions (31%) had almost perfect agreement (questions 3, 4, 5, 8, 18, 22, 27, 28, and 29), 15 questions (51.7%) had substantial agreement (questions 1, 2, 6, 7, 9, 12, 14, 15, 16, 17, 20, 21, 23, 25, and 26), 4 questions (13.8%) had moderate agreement (questions 10, 11, 19, and 24), and 1 question (3.5%) had reasonable agreement (question 13), as shown below.

**Table 3:** Agreement analysis of the 29 questions of the KORQ Questionnaire

Question	Kappa value	95% Confidence interval	*p* value	Question	Kappa value	95% Confidence interval	*p* value
1	0.767	0.72 - 0.91	<0.0001	19	0.550	0.47 -0.61	<0.0001
2	0.772	0.68 - 0.85	<0.0001	20	0.701	0.56 -0.74	<0.0001
3	0.817	0.66 - 0.90	<0.0001	21	0.771	0.72 - 0.90	<0.0001
4	0.857	0.81 - 0.90	<0.0001	22	0.943	0.91 - 0.99	<0.0001
5	0.902	0.86 - 0.95	<0.0001	23	0.742	0.69 - 0.81	<0.0001
6	0.688	0.35 - 0.79	<0.0001	24	0.510	0.46 - 0.61	<0.0001
7	0.729	0.72 - 0.91	<0.0001	25	0.649	0.49 - 0.73	<0.0001
8	0.823	0.64 - 0.99	<0.0001	26	0.735	0.64 - 0.79	<0.0001
9	0.679	0.51 - 0.94	<0.0001	27	0.865	0.81 - 0.99	<0.0001
10	0.473	0.33 - 0.53	<0.0001	28	0.817	0.77 - 0.85	<0.0001
11	0.471	0.42 - 0.52	<0.0001	29	0.817	0.79 - 0.91	<0.0001
12	0.770	0.69 - 0.99	<0.0001				
13	0.361	0.12 - 0.44	0.0003				
14	0.765	0.66 - 0.95	<0.0001				
15	0.645	0.58 - 0.79	<0.0001				
16	0.673	0.65 - 0.71	<0.0001				
17	0.740	0.72 - 0.78	<0.0001				
18	0.825	0.69 - 0.91	<0.0001				

KORQ= Keratoconus Outcomes Research Questionnaire.

## DISCUSSION

In patients with keratoconus, it is particularly important to measure the impact on QoL, because the disorder has an early onset, is progressive and chronic in nature, is associated with frequent changes in refractive prescription, can cause serious vision impairment, and typically requires treatment^(3,17)^. For this reason, an accurate measurement of QoL requires high-quality PRO measures. A variety of questionnaires have been used to evaluate QoL in keratoconus patients, but none has been developed specifically for this condition.

A previous study comparing the QoL questionnaires already used for keratoconus concluded that the KORQ was the only validated keratoconus-specific questionnaire and had the highest rating for psychometric properties among all the questionnaires^(18)^. It already contains a Rasch analysis that convert raw categorical data into linear interval-level data using a logarithmic transformation, which may improve the performance of the first-generation questionnaires^(19)^. However, although the KORQ had excellent psychometric properties and is currently the questionnaire recommended for measuring keratoconus outcomes, it measures only two domains of QoL: activity limitation and symptoms. Items on other QoL domains, including psychosocial well-being and inconvenience, are not included in the KORQ.

The present study indicates that the Portuguese translation and adaptation of the keratoconus function and symptom questionnaire, the KORQ, yielded a reliable tool, as evidenced by the high internal consistency of the answers obtained and the high correlation coefficients. In developing this tool, we followed the guidelines used previously in similar endeavors, including a scientifically rigorous process of translation and adaptation. Although the KORQ questionnaire comprises simple and direct questions, the use of two independent translators for each language translation was very useful because it allowed the multidisciplinary assessment panel to validate the questionnaire through comparisons and discussion. The inclusion of members of different areas of expertise in the assessment panel was also crucial, because it allowed comparisons from different points of view and solved discrepancies with the aims of consensus and proper adaptation.

It should be pointed out that a limitation of this study might be the small sample size. Although our sample size was in accordance with the recommendations of the cross-cultural adaptation guidelines^(15,16)^, a larger sample might have resulted in a much higher consistent agreement. Accordingly, our results demonstrate that this validated questionnaire is both reliable and reproducible and can be applied in future population-based studies to evaluate the impact of keratoconus on patient QoL.

In conclusion, the KORQ is a meticulously developed and Rasch analysis-tested and scaled instrument that fulfills the need for a disease-specific instrument capable of measuring outcomes of treatment and intervention in patients with keratoconus. The translation and validation into the Portuguese language presented herein is a powerful tool that will contribute to assessing the impact of this important ocular condition on the QoL of patients in Brazil.
